# Contributing Risk Factors for Orthopedic Device Related Infections in Sina Hospital, Tehran, Iran

**Published:** 2011-02-01

**Authors:** A Hadadi, M J Zehtab, H Babagolzadeh, H Ashraf

**Affiliations:** 1 Department of Infectious Disease, Research Development Center, Sina Hospital, Iranian Research Center for HIV/AIDS, Tehran University of Medical Sciences, Tehran, Iran; 2 Department of Orthopedic Surgery, Sina Hospital, Tehran University of Medical Sciences, Tehran, Iran; 3 General Practitioner, Sina Hospital, Tehran University of Medical Sciences, Tehran, Iran; 4 Department of Cardiology, Research and Development Center, Sina Hospital, Tehran University of Medical Sciences, Tehran, Iran

**Keywords:** Orthopedic devices, Infection, Implant surgery, Microorganisms

## Abstract

**Background:**

In spite of decreasing incidence of orthopedic device-related infections to 1%, nowadays, device-related infection still remains a diagnostic, therapeutic and cost-related problem. The objective of this study is to evaluate the contributing risk factors for orthopedic device-related infections in Sina Hospital, Tehran, Iran.

**Methods:**

Three hundred and thirty patients who underwent orthopedic device implantation from 2002-2006 were enrolled; among them, 110 patients were complicated with infection. Descriptive and logistic regression analyses were performed to determine the risk factors for device related infections.

**Results:**

Patients with infection were older compared to those without infection. The Staphylococcus aureus was the commonest organism. A correlation was observed between wound infection and external fixation, an underlying health condition, and addiction which were independent risk factors for a device related infection.

**Conclusion:**

Orthopedic device–related infection puts a great financial burden on patients and hospital resources and could lead to morbidity and mortality in patients. So, appropriate pre and postoperative wound care for dirty wounds, especially when external fixators are used, and in patients with poor conditions or addiction should be done with more caution.

## Introduction

The number of elderly and trauma patients requiring joint replacement or fixation devices is steadily increasing. The risk of infectious complications associated with orthopedic devices has been decreased during the past 2 decades, with development of sophisticated preventive strategies. Infections associated with prosthetic joints occur less frequently than aseptic failures, but they represent the most devastating complications with high morbidity and substantial costs.[1] Overall, about 5% of the internal fixation devices become infected.[2] The incidence of infection after the internal fixation of closed fractures is generally lower (0.5–2%), whereas the incidence may exceed 30% after the fixation of open fractures.[3][4][5]

It is expected that the incidence of orthopedic device–related infections (ODRIs) and the absolute number of patients with such infections will further increase due to better detection methods, the growing number of implanted prostheses in the aging population, and the increasing residency time of prostheses, which are at a continuous risk of infection during their implanted lifetime.[6] Furthermore, due to the scarcity of infections per institution, randomized controlled clinical trials are hampered and the treatment of such infections is poorly standardized. Therefore, ODRI is still a problem for both the patient and the surgeon, especially in developing countries, where it has a great financial burden on the patient and hospital resources.

The objective of this retrospective study was to identify the variables that contribute to infection in orthopedic device surgeries in a public hospital and to evaluate microbiologic aspects and therapeutic measures.

## Materials and Methods

In the present case-control study, we have evaluated 110 subjects with the diagnosis of orthopedic implant infection during the first year after their surgery who needed hospitalization, and 220 subjects with orthopedic device were included in the control group. This study was based on the data collected from the records of patients who underwent orthopedic device implantation in Sina Hospital affiliated to Tehran University of Medical Sciences, Tehran, Iran during the years 2002-2006.

The medical records, including the associated factors of ODRI were reviewed, and then were compared between patients with and without infection. The basic clinical information on patient demographics, underlying disease status (use of immunosuppressive drugs, co-existing malignancy, chronic diseases and diabetes mellitus), duration of procedure, smoking or addiction history, use of prophylactic antibiotics, wound class, timing of surgery (emergency or elective), and type of implanted device were collected.

The diagnosis of infection was based on clinical and microbiological reports. Infections were classified into two stages, i.e., early (less than 2-4 weeks), and late (within one month to one year) infections.[7] The study protocol was approved by the Research Ethics Committee of Tehran University of Medical Sciences. The present study was conducted in conformity with the Helsinki declaration.

The statistical analyses were performed using SPSS software (SPSS, Chicago, IL, USA; Version 17). The continuous variables were shown as means±standard deviation. The univariate analysis of the categorical outcome (development of ODRI) and each individual associated factor was carried out using Chi-Square test. Student’s t test was used to compare parametric quantitative variables and Chi-Square or Fisher’s Exact test to compare the proportions. Odds ratios (OR) and 95% confidence intervals (95% CI) were calculated. Then, in a multiple logistic regression, we explored the effect of independent variables for ODRI by adding predictors in a stepwise manner to examine if the factor was associated with the development of infection while adjusting for potential confounders and effect modifiers. p<0.05 was considered statistically significant.

## Results

Three hundred and thirty orthopedic surgical patients were included in this study, among them, 110 patients were complicated with a device-related infection. The demographic and clinical characteristics of the patients are shown in Table 1. Patients with infection were older than those without infection (40.9±1.8 vs. 35.6±1.1, p=0.010), while there was no difference concerning male to female ratio between two groups (84/26 vs. 176/44 p= 0.476). Internal bone fixators were the most frequent devices which were used in 79.7% (n=263) of patients. When the subjects were grouped by wound classification, there were 23.6% clean, 1.8% clean-contaminated, and 74.5% dirty wounds in patients with ORDI, while among patients free of infection, these percentages were 75.5%, 0.5% and 24.1%, respectively (Table 2). Prophylactic antibiotics of the first generation cephalosporins, i.e., cefazoline were administered for all patients. Most of patients required elective surgery (N=317; 96.1%) and in all cases, the procedure time was more than 2 hours. Purulent discharge, swelling and pain were the most frequent clinical presentations of the subjects with ORDI (in 101, 37, and 17 cases, respectively); followed by fever (7 cases), formation of sinus tract and device loosening (each in one case). Out of 110 infected cases, the device was removed in 44 cases (21.8%) while the rest of the patients were treated with intravenous antibiotics and multiple debridements.

The microbiological report of 24 patients with ODRI was not available in their medical records. Of the 74 patients with microbiological positive wound infections, 2 (2.7%) had polymicrobial infections. The most frequently isolated bacteria were Staphyloccocus aureus (41 cases), Gram negative bacilli (25 cases), coagulase negative Staphyloccoci (4 cases) and Enterococcus spp (2 cases). There were 60 cases (54.5%) of infection in early and 50 cases (45.5%) in late stages. Among the patients with Gram positive isolates, 59.2% had early and 40.8% had late infection, while in the ones with Gram negative bacilli, these rates were 56% and 44%, respectively. About 76.7% of early infections and 78% of late infections were of Gram positive strains.

**Table 1:** Basic characteristics of orthopedic patients underwent device implantation.

****	**CaseN=110**	**ControlN=220**
Mean age in years (SE)	40.96 (1.79)	35.65 (1.15)
Gender (M/F)	84/26	176/44
Wound class N (%)		
Clean	26 (23.6)	166 (75.5)
Clean-contaminated	2 (1.8)	1 (0.5)
Dirty	82 (74.5)	53 (24.1)
Device (N)		
Fixator (External/ Internal) a	(28/83)	(16/196)
Prosthesis (hip/knee)	(1/2)	(8/10)
Timing of surgery N (%)		
Emergency	2 (1.8)	1 (0.5)
Elective	108 (98.2)	219 (99.5)
Type of anesthesia N (%)		
Local	50 (45.5)	94 (42.7)
General	56 (50.9)	122 (55.5)
Both	4 (3.6)	4 (1.8)

^a^ Twelve patients had infections of both internal and external fixators together.

**Table 2:** Association between orthopedic device-related infections and investigated factors, according to univariate logistic regression analysis.

**Risk factors**	**No. (%) of patients**		**OR**	**95% CI**	**P value**
	**With ODRI a (N=110)**	**Without ODRI a (N=220)**			
Age (years, mean)	40.96 (1.79)	35.65 (1.15)	1.02	1.0–1.03	.0120
Dirty wound class procedure	82 (74.5)	53 (24.1)	9.22	5.43–15.66	<0.001
Motor-vehicle-related trauma	83 (75.5)	129 (58.6)	2.69	1.30–3.51	0.003
External Fixator	24 (21.8)	6 (2.7)	4.35	22.24–8.47	<0.001
Underlying diseases	13 (11.8)	2 (0.9)	14.61	3.23–65.98	<0.001
Addiction	15 (12.6)	7 (3.2)	4.81	1.89–12.17	<0.001

^a^ ODRI, orthopedic device related infection

The risk factors associated with increased ORDI rates revealed by univariate logistic regression included higher age, motor-vehicle-related surgery, dirty procedures, surgery with external fixation, underlying health conditions and addiction (Table 2). Sex (OR= 1.2, 95% CI=0.71–2.15), use of prosthesis (OR=0.31, 95% CI=0.09 –1.09) or implant (OR=0.34, 95% CI=0.19 – 0.62), and type of anesthesia (p=0.5) were not related to ORDI. In multi-variate analyses, having a dirty wound, procedures with external fixation, underlying conditions and addiction were independent risk factors for ORDI (Table 3). A multi-variate analysis to evaluate the risk factors for Gram-positive infections revealed that procedures for motor-vehicle-related trauma were related to these infections (OR=8.69, 1.0 p=0.040).

**Table 3:** Independent risk factors for device-related infections in orthopedic patients from multiple regression models.

	**OR**	**95% CI**	**P value**
Dirty wound class Procedure	9.96	4.96–20.06	<0.001
External fixator	7.35	2.54–21.28	<0.001
Underlying diseases	16.39	3.08–83.33	0.001
Addiction	4.081	1.31–12.82	0.015

## Discussion

The current study reveals special issues contributing to the risk factors of ORDIs in orthopedic patients at a leading teaching hospital in Tehran, Iran. The inherent risk of infection associated with the implantation of foreign devices in the human body increases in orthopedic surgery by several factors. A main factor is that the dead space is always present around the implanted device,[8] in which hematoma increases the risk of infection through several mechanisms. First, a hematoma is an appropriate medium for bacterial growth. Previous studies have proposed that antibiotics administered postoperatively do not penetrate hematomas easily and may not reach a clinically effective concentration in the hematoma.[9] Second, the presence of a hematoma can also decrease the ability of normal defense mechanisms by devascularization of the tissue near the wound. The presence of a hematoma can also prevent the entry of antibiotics into the surrounding tissues.[8] Another factor is the inherently low blood flow to the cortical bone [10] which is compromised to a greater extent by the surgical techniques required for device implantation. The reaming of the bone results in death of the tissue in the immediate area and further decreases the blood supply and an increased presence of a dead bony tissue.[8]

In addition to the inherent risks of infection associated with orthopedic devices, many intrinsic, extrinsic risk factors could involve in the pathogenesis of ODRIs and orthopedic surgical site infections (SSIs). The intrinsic factors related to patient status include aging, patients’ health condition, nutritional status, obesity, additional nosocomial infections, long preoperative stay and corticosteroid therapy.[11][12][13][14] Patients with a history of trauma have a higher incidence of wound infection., The problems with healing of fractures, postsurgical sepsis, and nutritional status are also important factors in this situation.[15][16] The major surgical risk factors include the number of operations, dirty and contaminated wounds, antibiotic prophylaxis, postoperative hematoma formation, persistent drainage (after 48 h), and type of anesthesia.[13][14][17]

A recent investigation of risk factors for SSI among teaching hospitals in Tehran revealed that the risk of SSI was increased by age in persons older than 60 years (OR=3·9;), diabetes mellitus (OR=4·9;), smoking (OR=3·1;), obesity (OR=4·1;) and wound drain (OR=2·2; p<0·0001). There were significant statistical differences during the anesthesia (131·6 vs. 177 minutes, p<0·001) and the surgery (99 vs. 140·5 minutes) between patients with/without SSI.[18] These factors should be considered by the surgeon when he is considering a surgery and is planning postoperative care for the patient.

The contributing factors of ODRI, operable in our study, were a dirty wound, procedures during an external fixation, the underlying health condition and addiction. These findings suggest that it would be worthy to review and modify the protocol for postoperative wound care for this group of patients. Although the usefulness of the traditional wound classification has been doubted,[19] as we have shown, it was an important predictor of ODRI and this finding was confirmed by our study.

Emergency surgery for motor-vehicle-related trauma was not an independent risk factor for ODRI in this study. However, considering the fact that orthopedic patients have been reported to be more prone to infections amongst patients with trauma,[20] motor-vehicle associated collisions, as a main cause of trauma-related surgical orthopedic patient hospitalizations, highlight the necessity in these cases of obtaining appropriate wound cultures before operation and the judicious use of prophylactic and therapeutic antibiotics.

In our study, the advanced age was responsible for infections (although it was not shown to be an independent contributing factor) as reported in other studies, as well.[21][22] A possible factor contributing to this may be the poorer immune, nutritional status of these patients or age-related differences in the severity of trauma or type of procedure. Also, Scott et al.[23] reported that older patients with lower albumin were associated with SSI in a study of 9016 surgical patients in New York.

According to the present results, positive culture was seen in the majority of the studied patients with available culture reports (86%), while in the study of Gomez et al.,[24] the reported positive cultures were 60%. The finding of Zimmeli et al.[25] was close to us, with a reported value of 89%. The bacterial spectrum associated with orthopedic devices in our study consisted mainly of Staphylococcus species, which strongly implicates the intraoperative contamination scenario[26] and assume that these are the main nosocomial pathogens in our operating room. The present findings are in agreement with the extensive study of Arciola et al. and the earlier culturing results of orthopedic implants.[27] As expected, and in line with the finding of Gomez et al.,[24] a dominant part was consisted of gram-positive positive cocci (66.2%), although numerous occurrences of Gram-negative bacterial were also identified. This was in contrast to a previous study in Iran [28] with a different incidence rate for Gram-positive and Gram-negative bacterial isolates as 33.5% vs. 64.5%, respectively, probably due to different nosocomial pathogens present in our hospital. Besides, the rate of Gram-negative isolates in their study was higher compared to coagulase negative Staphyloccoci and Enterococcus spp, since, the majority of implant infections were late onsets (67%), reflecting that Gram-negative isolates appear to play a significant role in the pathogenesis of late-onset postoperative infections in this study. In addition, the anaerobic bacteria were not isolated in the present study because we do not have an appropriate culture for anaerobes. We assume that some negative culture reports of our patients are attributed to anaerobes.

The treatment of ODRIs most frequently includes long-term antimicrobial treatments and the removal of the implants. In our study, devices were removed in 21.8% of cases while the rest of the patients were treated with intravenous antibiotics and multiple debridements. A recent evidence from observational trials[21],[29] and one randomized clinical trial [30] indicated that a subset of patients can be successfully treated by debridement and long-term antimicrobial therapy with the retention of the implant. It is stated that patients eligible for such a treatment must meet the following criteria: Acute infection defined as signs and symptoms lasting <14–28 days, an unambiguous diagnosis based on histopathology and microbiology, a stable implant and good quality of bone stock, and the susceptibility of the microorganism to an effective orally available antimicrobial agent.[31]

There are limitations in this study that should be taken into account when interpreting the findings. As all of the patients were receiving antibiotics postoperatively, this could not be used as an indicator of ODRI and thus it is not possible to clearly define appropriate antibiotic prophylaxis. The study also did not evaluate some intrinsic factors such as the patient’s nutritional status and special concomitant diseases that can be involved with ORDI.

The results of this study emphasize the need to account for local factors when assessing ODRI risk. Appropriate pre and postoperative wound care for dirty wounds especially when external fixators are used and in patients with poor condition or addiction should be done with more caution. The obtained data confirm the necessity of a review and modification of the protocol for wound care in this group of patients and may even require a special protocol.
